# Addiction of Merkel cell carcinoma to MUC1-C identifies a potential new target for treatment

**DOI:** 10.1038/s41388-022-02361-3

**Published:** 2022-06-10

**Authors:** Yoshihiro Morimoto, Atsushi Fushimi, Nami Yamashita, Masayuki Hagiwara, Atrayee Bhattacharya, Jingwei Cheng, Thomas C. Frost, Rehan Ahmad, Tatsuaki Daimon, Lei Huang, Tsuyoshi Hata, Hidekazu Takahashi, Masaaki Yamamoto, Yozo Suzuki, James A. DeCaprio, Donald Kufe

**Affiliations:** 1 Dana-Farber Cancer Institute, Harvard Medical School, Boston, MA USA; 2 Department of Molecular, Cellular and Biomedical Sciences University of New Hampshire, Durham, NH USA; 3 Department of Histoembryology, Genetics and Developmental Biology, Key Laboratory of Cell Differentiation and Apoptosis of Chinese Ministry of Education, Shanghai Key Laboratory of Reproductive Medicine, Shanghai Jiao Tong University School of Medicine, Shanghai, P. R. China; 4 Present Address: Department of Urology, Keio University School of Medicine, Shinjuku-ku, Tokyo, 160-8582 Japan; 5 Present Address: Department of Gastroenterological Surgery, Graduate School of Medicine, Osaka University, Suita, Osaka 565-0871 Japan

**Keywords:** Skin cancer, Oncogenes, Cancer stem cells, Cancer therapy

## Abstract

Merkel cell carcinoma (MCC) is an aggressive malignancy with neuroendocrine (NE) features, limited treatment options, and a lack of druggable targets. There is no reported involvement of the MUC1-C oncogenic protein in MCC progression. We show here that MUC1-C is broadly expressed in MCCs and at higher levels in Merkel cell polyomavirus (MCPyV)-positive (MCCP) relative to MCPyV-negative (MCCN) tumors. Our results further demonstrate that MUC1-C is expressed in MCCP, as well as MCCN, cell lines and regulates common sets of signaling pathways related to RNA synthesis, processing, and transport in both subtypes. Mechanistically, MUC1-C (i) interacts with MYCL, which drives MCC progression, (ii) is necessary for expression of the OCT4, SOX2, KLF4, MYC, and NANOG pluripotency factors, and (iii) induces the NEUROD1, BRN2 and ATOH1 NE lineage dictating transcription factors. We show that MUC1-C is also necessary for MCCP and MCCN cell survival by suppressing DNA replication stress, the p53 pathway, and apoptosis. In concert with these results, targeting MUC1-C genetically and pharmacologically inhibits MCC self-renewal capacity and tumorigenicity. These findings demonstrate that MCCP and MCCN cells are addicted to MUC1-C and identify MUC1-C as a potential target for MCC treatment.

## Introduction

Merkel cell carcinoma (MCC) is an aggressive and recalcitrant neuroendocrine (NE) cancer with no effective targeted therapies [1, 2]. One form of MCC, accounting for ~60% of tumors, is driven by clonal integration of Merkel cell polyomavirus (MCPyV) DNA into the MCC cell genome [2]. The other nonviral form of MCC, induced by chronic UV damage, harbors a high mutational burden associated with inactivation of p53 and RB [2, 3]. In MCPyV-positive MCC (MCCP) cells, which often express wild-type RB and p53, the MCPyV large T (LT) antigen binds directly to RB and inhibits its tumor suppressor functions. In contrast to LT, which is frequently expressed as a truncated and mutated protein, the MCPyV small T (ST) antigen is usually wild type and essential for MCPyV-induced transformation [2]. MCPyV ST forms a complex with the MYC paralog MYCL (L-MYC) and EP400 to induce downstream target genes which encode in part the E3 ubiquitin ligase MDM2 that promotes p53 degradation [4, 5]. Among the common chromosomal alterations in MCCs, amplification of 1p (cluster 4), which includes the *MYCL* locus, is found more commonly, but not exclusively, in MCPyV-negative MCC (MCCN) tumors, supporting the role of MYCL in driving MCC progression [6]. Of interest, MCCP and MCCN tumors are both largely refractory to treatment with genotoxic anti-cancer agents, such as etoposide and carboplatin [1]. Responsiveness of MCCP and MCCN tumors to immune checkpoint inhibitors (ICIs) is also independent of MCPyV status [1]. Moreover, UV exposure, the presence of MCPyV, and the mutational status of RB and p53 are not significantly associated with clinical relapse after initial therapy [6], indicating that other effectors may be of importance in MCC progression.

The *MUC1* gene appeared in mammals to protect epithelia from inflammation and damage induced by exposure to the external environment [7, 8]. *MUC1* encodes a protein that undergoes autoproteolyic cleavage into N-terminal (MUC1-N) and C-terminal (MUC1-C) subunits [7, 8]. In response to loss of epithelial homeostasis, (i) MUC1-N is shed from the cell surface into the protective mucous barrier, and (ii) the transmembrane MUC1-C subunit activates inflammatory, proliferative, and remodeling pathways associated with the process of wound healing [8]. However, given this capacity to restore homeostasis, the appropriation of prolonged MUC1-C activation resulting from chronic infections and repetitive cycles of damage and repair promotes carcinogenesis [8–10]. As a result, MUC1-C is typically overexpressed in carcinomas and contributes to diverse hallmark traits of the cancer cell [7–9]. Along these lines, MUC1-C is imported into the nucleus by interacting with importin-β and the nuclear pore complex nucleoporin 62 (NUP62), NUP358, NUP214, and NUP88 proteins at the cytoplasmic face [8]. MUC1-C induces the epithelial-mesenchymal transition and epigenetic reprogramming with the repression of tumor suppressor genes [8, 11, 12]. MUC1-C also induces the remodeling of chromatin necessary for lineage plasticity and the cancer stem cell (CSC) state [8, 13, 14]. Lineage plasticity is linked to DNA damage resistance and, in this context, MUC1-C protects against the effects of genotoxic anti-cancer agents in part by (i) suppressing activation of the p53 pathway and the induction of apoptosis [8, 15, 16], and (ii) integrating chromatin remodeling with the repair of DNA damage in carcinoma cells [17, 18]. These findings have supported a role for MUC1-C in driving epigenetic alterations that have been subverted by cancer cells to promote dedifferentiation, lineage plasticity, and treatment resistance.

There is no reported involvement of MUC1-C in MCC progression. Indeed, little is known about the expression of MUC1 in MCCP and MCCN tumors [19]. We show that MUC1 is expressed in both MCCP and MCCN tumors. We also show that silencing MUC1-C in MCCP and MCCN cells suppresses expression of (i) MYCL, (ii) pluripotency factors, and (iii) NE differentiation transcription factors (TFs). In addition, we demonstrate that MUC1-C is necessary for suppressing DNA replicative stress, DNA damage, and apoptosis. Consistent with these results, targeting MUC1-C genetically and pharmacologically inhibits MCC cell self-renewal capacity and tumorigenicity. Our findings demonstrate that MCC cells are addicted to MUC1-C, as widely defined by dependence on a gene for survival [20, 21], and identify a potential new target for advancing MCC treatment.

## Results

### MUC1 is widely expressed in MCC tumors and cell lines

**Fig. 1:**
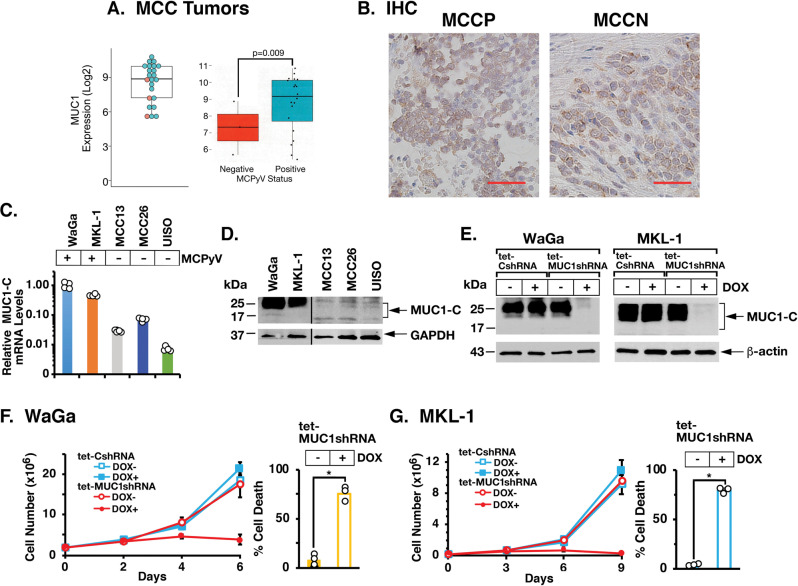
Expression of MUC1 in MCC tumors and cell lines. **A** Analysis of MUC1 expression using RNA-seq datasets derived from 23 MCC tumors (left) and from 55 MCCP and MCCN tumors (right). **B** IHC staining of MUC1-C in MCCP (left) and MCCN (right) tumor cells. **C** The indicated MCCP and MCCN cell lines were analyzed for MUC1-C mRNA levels by qRT-PCR using primers listed in Supplementary Table S1. The results (mean ± SD of four determinations) are expressed as relative mRNA levels compared to that obtained for WaGa cells (assigned a value of 1). **D** Lysates from the indicated MCCP and MCCN cells were immunoblotted with antibodies against MUC1-C and GAPDH. **E** WaGa (left) and MKL-1 (right) cells expressing a tet-CshRNA or a tet-MUC1shRNA were treated with vehicle or 500 ng/ml DOX for 4 and 6 days, respectively. Lysates were immunoblotted with antibodies against the indicated proteins. **F**, **G** WaGa/tet-CshRNA or WaGa/tet-MUC1shRNA (**F**) and MKL-1/tet-CshRNA or MKL-1/tet-MUC1shRNA (**G**) cells treated with vehicle or 500 ng/ml DOX for 6 and 9 days, respectively, were analyzed for proliferation (left) and percentage cell death (right) by trypan blue staining. The results are expressed as the mean ± SD of three separate determinations.

Analysis of the GSE50451 microarray RNA expression dataset from 23 MCC tissues [22] demonstrated that MUC1 mRNA levels are uniformly detectable in these tumors (Fig. 1A, left). Analysis of RNA-seq data from an additional 55 MCCs further revealed that MUC1 expression is significantly higher in MCCP, as compared to MCCN, tumors (Fig. 1A, right). Previous work showed that MUC1-N is expressed in MCC tumor tissues [19]. *MUC1* also encodes the oncogenic MUC1-C subunit [8], which we found by immunohistochemistry (IHC) is expressed in MCCP and MCCN tumor, and not surrounding normal, tissues (Fig. 1B, left and right; Supplementary Fig. S1A). WaGa and MKL-1 MCCP cells more closely model MCC tumors than the variant MCC13, MCC26, and UISO MCCN cell lines [22]. We found that MUC1 mRNA levels in WaGa and MKL-1 MCCP cells are significantly higher than that in the MCCN cells (Fig. 1C). Expression of the MUC1-C subunit as the N-glycosylated 25–20 kDa and unglycosylated ~17 kDa proteins was also substantially higher in the MCCP cells (Fig. 1D). To assess the potential significance of these results, we established WaGa cells stably expressing a tet-inducible control shRNA (tet-CshRNA) or a tet-MUC1shRNA. Treatment with doxycycline (DOX) was associated with downregulation of MUC1-C in WaGa/tet-MUC1shRNA, and not WaGa/tet-CshRNA, cells (Fig. 1E, left). Similar results were obtained from DOX-treated MKL-1/tet-CshRNA and MKL-1/tet-MUC1shRNA cells (Fig. 1E, right). Unexpectedly during the course of these experiments, we found that silencing MUC1-C in WaGa (Fig. 1F, left and right) and MKL-1 (Fig. 1G, left and right) cells rapidly results in inhibition of proliferation and induction of cell death. As a control, rescue of MUC1-C expression in DOX-treated WaGa/tet-MUC1shRNA and MKL-1/tet-MUC1shRNA cells attenuated the loss of survival (Supplementary Fig. S1B, C).

### MUC1-C regulates MCCP cell transcriptomes

**Fig. 2:**
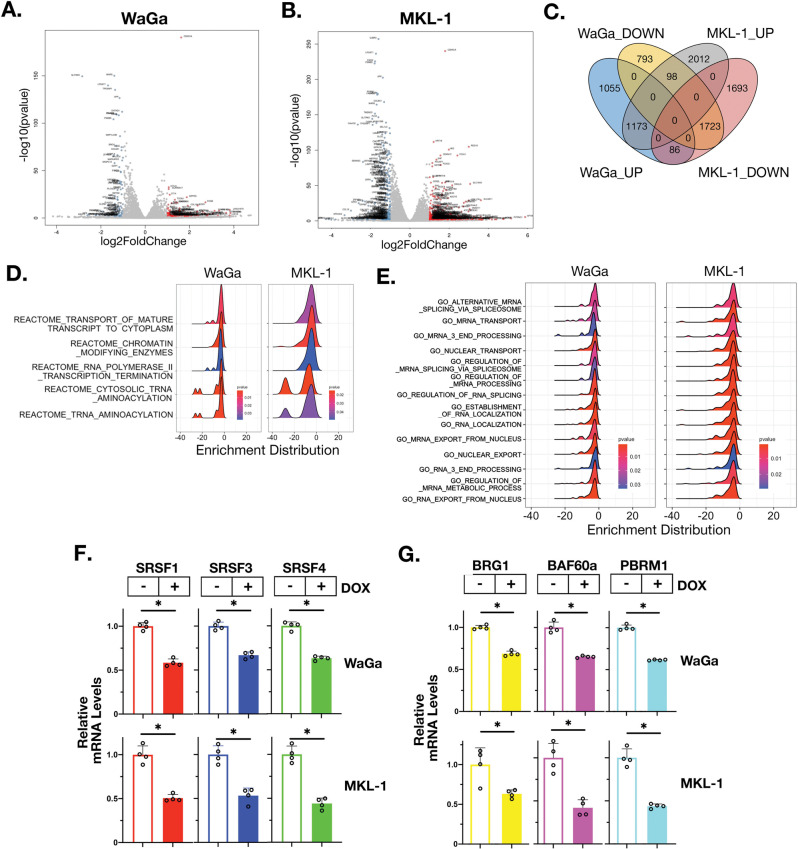
MUC1-C regulates common gene signatures in WaGa and MKL-1 cells. **A**, **B** RNA-seq was performed in triplicate on WaGa/tet-MUC1shRNA (**A**) and MKL-1/tet-MUC1shRNA (**B**) cells treated with vehicle or DOX for 3 days. The datasets were analyzed for effects of MUC1-C silencing on down- and upregulated genes as depicted by the Volcano plots. **C** Overlap of MUC1-C-driven down- and upregulated genes in WaGa and MKL-1 cells. **D**, **E** Effects of MUC1-C silencing in WaGa (left) and MKL-1 (right) cells on REACTOME DN (**D**) and GO DN (**E**) gene signatures. **F**, **G** qRT-PCR analysis of selected common downregulated SRSF (**F**) and SWI/SNF complex (**G**) genes in WaGa/tet-MUC1shRNA (upper panels) and MKL-1/tet-MUC1shRNA (lower panels) cells treated with vehicle or DOX for 3 and 5 days, respectively. The results (mean ± SD of four determinations) are expressed as relative mRNA levels compared to that obtained for vehicle-treated cells (assigned a value of 1).

Given the dependence of MCCP cells on MUC1-C for growth and viability, we performed RNA-seq studies to assess the effects of silencing MUC1-C on gene expression patterns. Volcano plots of MUC1-C-silenced WaGa and MKL-1 cells revealed marked changes in gene repression and induction (Fig. 2A, B). Among these, we identified 1723 downregulated genes and 1173 upregulated genes common to both cell lines (Fig. 2C). In concert with these results, analysis by Gene Set Enrichment Analysis (GSEA) using the REACTOME collection demonstrated that silencing MUC1-C in WaGa and MKL-1 cells associates with the induction of gene signatures related to transcription and mRNA processing (Fig. 2D, left and right). Further analysis of the GO collection confirmed that silencing MUC1-C significantly correlates with the regulation of RNA synthesis, processing, and transport (Fig. 2E, left and right). Among these genes, we identified members encoding (i) a family encoding serine/arginine-rich splicing factors that play roles in RNA metabolism, including alternative splicing and intron retention [23, 24] (Fig. 2F), and (ii) the SWI/SNF BAF and PBAF chromatin remodeling complexes, which have been linked to MUC1-C-induced progression of prostate carcinoma cells [14, 25] (Fig. 2G).

### MUC1-C dependency of MCC26 MCCN cells

**Fig. 3:**
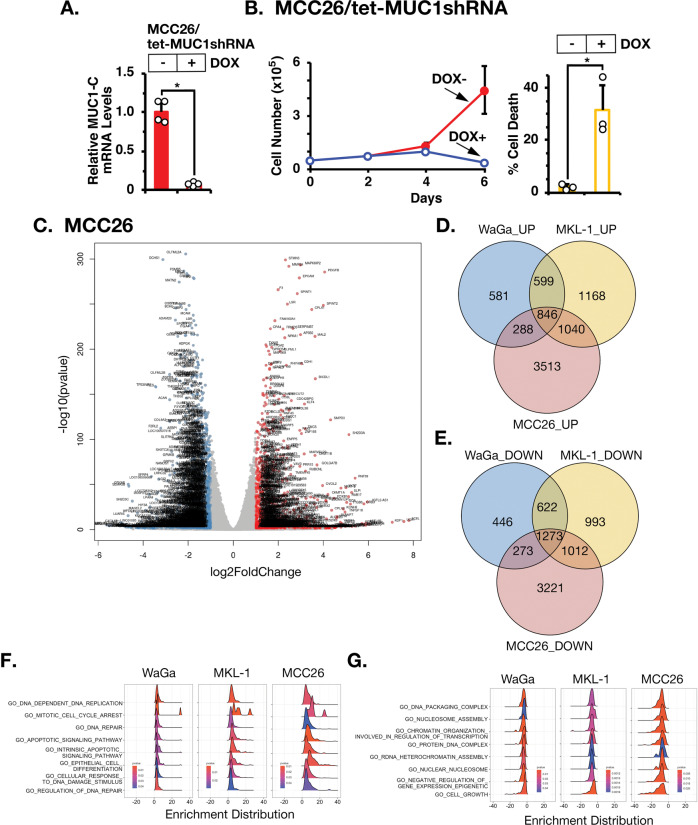
Effects of silencing MUC1-C in MCC26 MCCN cells. **A** MCC26/tet-MUC1shRNA cells treated with vehicle or DOX for 6 days were analyzed for MUC1-C mRNA levels by qRT-PCR. The results (mean ± SD of four determinations) are expressed as relative MUC1-C mRNA levels compared to that obtained for vehicle-treated cells (assigned a value of 1). **B** MCC26/tet-MUC1shRNA cells treated with vehicle or DOX for 6 days were analyzed for growth (left) and percentage cell death (right). The results are expressed as the mean ± SD of three separate determinations. **C** RNA-seq was performed in triplicate on MCC26/tet-MUC1shRNA cells treated with vehicle of DOX for 4 days. The datasets were analyzed for effects of MUC1-C silencing on down- and upregulated genes as depicted by the Volcano plots. **D**, **E** Venn diagrams of common upregulated (**D**) and downregulated (**E**) genes in WaGa/tet-MUC1shRNA, MKL-1/tet-MUC1shRNA, and MCC26/tet-MUC1shRNA cells treated with DOX for 3, 5, and 4 days, respectively. **F**, **G**. Common upregulated (**F**) and downregulated (**G**) pathways in WaGa/tet-MUC1shRNA, MKL-1/tet-MUC1shRNA, and MCC26/tet-MUC1shRNA cells.

The findings that MUC1-C levels are higher in MCCP, compared to that in MCCN tumors and cell lines invoked the possibility that MCPyV status dictates MUC1-C expression and MUC1-C-induced gene signatures. Notably, however, we found that inducible downregulation of LT and ST has no apparent effect on MUC1-C levels (Supplementary Fig. S2A). In addition, silencing MUC1-C in WaGa cells had little if any effect on LT and ST levels (Supplementary Fig. S2B). Whereas these results do not preclude the possibility that MCPyV drives MUC1-C expression by other mechanisms, they invoked the prospect that MUC1-C dependency in MCC is dictated by MCPyV status. Accordingly, we silenced MUC1-C in MCC26 MCCN cells (Fig. 3A). Consistent with the responses of WaGa and MKL-1 cells, we found that MCC26 cells are also dependent on MUC1-C for proliferation and survival (Fig. 3B, left and right). The MUC1shRNA targets sequences that encode the MUC1-C extracellular domain. As a control, we generated an anti-sense oligonucleotide (ASO) against sequences encoding the MUC1-C intracellular domain and confirmed dependence on MUC1-C for survival (Supplementary Fig. S3A–C). RNA-seq studies performed on MCC26 cells further demonstrated the global effects of MUC1-C silencing on gene activation and repression (Fig. 3C). In addition, analysis of the WaGa, MKL-1, and MCC26 cell datasets identified (i) 846 common upregulated genes that included *P21*, *FAS*, *CDH1*, and *FBXW7* (Fig. 3D), and (ii) 1273 common downregulated genes, among which were *CTNNB1* and *BMI1* (Fig. 3E), indicating that MUC1-C drives similar gene signatures in MCCP and MCCN cells. Along these lines, we found concordance of MUC1-C upregulated and downregulated GO gene signatures associated with DNA replication, DNA damage repair, and epigenetic processes in WaGa, MKL-1, and MCC26 cells (Fig. 3F, G).

### MUC1-C drives pluripotency and lineage dictating TFs in MCC cells

**Fig. 4:**
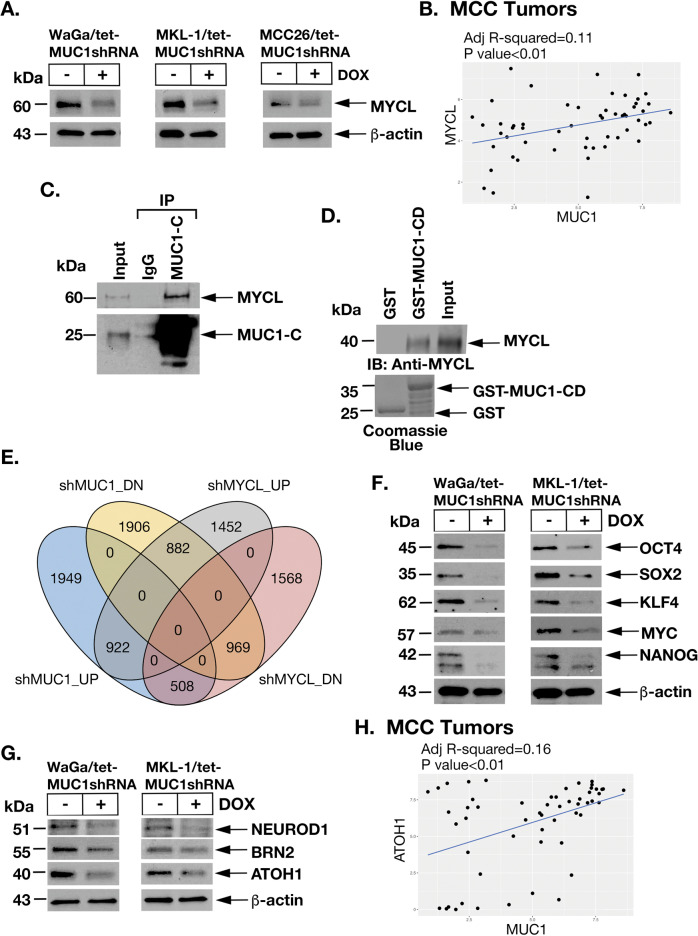
MUC1-C induces the MYCL, pluripotency, and NE lineage dictating TFs in MCC cells. **A** Lysates from WaGa/tet-MUC1shRNA, MKL-1/tet-MUC1shRNA, and MCC26/tet-MUC1shRNA cells treated with vehicle or DOX for 4, 6, and 6 days, respectively, were immunoblotted with antibodies against the indicated proteins**. B** Scatter plot of the correlation between MUC1 and MYCL expression in MCC tumors. **C** Nuclear lysates from WaGa cells were precipitated with anti-MUC1-C and a control IgG. Input lysate and the precipitates were immunoblotted with antibodies against the indicated proteins**. D** GST and GST-MUC1-CD(FL; 1–72 aa) were incubated with purified full-length MYCL(1–364). The adsorbates and input were immunoblotted with anti-MYCL. Input of the GST proteins was assessed by Coomassie blue staining. **E** Venn diagram of common upregulated and downregulated genes in MKL-1/tet-MUC1shRNA and MKL-1/tet-MYCLshRNA cells treated with DOX for 5 days. **F**, **G** Lysates from WaGa/tet-MUC1shRNA (left) and MKL-1/tet-MUC1shRNA (right) cells treated with vehicle or DOX for 4 and 6 days, respectively, days were immunoblotted with antibodies against the indicated proteins**. H** Scatter plot of the correlation between MUC1 and ATOH1 expression in MCC tumors.

The MYC family of TFs includes MYC (c-MYC), MYCL (L-MYC), and MYCN (N-MYC) [26]. A frequent chromosomal change in MCCP and MCCN tumors is an amplification of the *MYCL* locus [6]. Overexpression of MYCL promotes MCC cell survival [4]. To our knowledge, there is no recognized relationship between MUC1-C and MYCL. We found that silencing MUC1-C partially suppresses MYCL expression in WaGa, MKL-1, and MCC26 cells (Fig. 4A). In support of these results, MUC1 was significantly correlated with MYCL expression (Fig. 4B) and activation of the HALLMARK MYC TARGETS V1 gene signature (Supplementary Fig. S4A) in MCC tumors. MUC1-C localizes to the nucleus in human cancer cells, where it interacts with TFs and effectors of epigenetic reprogramming and chromatin remodeling complexes [8]. Among TFs, MUC1-C induces MYC (c-MYC) in certain cancer cells and interacts with MYC in promoting the activation of MYC target genes [27]. Here, we found that MUC1-C interacts with nuclear MYCL in MCC cells (Fig. 4C). In vitro binding studies further demonstrated that the MUC1-C cytoplasmic domain (MUC1-CD) interacts directly with MYCL (Fig. 4D), supporting a potential MUC1-C→MYCL signaling pathway that involves MUC1-C induced MYCL expression and the MYCL transactivation function. In addressing this notion, we compared MUC1-C and MYCL transcriptomes in MKL-1 cells and found a significant overlap of 922 upregulated and 969 downregulated genes (Fig. 4E and Supplementary Fig. S4B), which included pluripotency and NE differentiation TFs, among others. In this regard, silencing MUC1-C in WaGa and MKL-1 cells was associated with suppression of (i) the Yamanaka OCT4, SOX2, KLF4, and MYC (OSKM) pluripotency factors that collectively dedifferentiate fibroblasts to induced pluripotent stem cells [28], and (ii) NANOG, another core pluripotency factor that is essential for dedifferentiation of primitive stem cells [29] (Fig. 4F, left and right). We also found in MCC tumors that MUC1 significantly associates with activation of the BENPORATH ES 1 embryonic stem cell-like gene signature (Supplementary Fig. S4C) [30], indicating that MUC1-C drives the MCC CSC state. The OSKM factors function as pioneer TFs in promoting chromatin accessibility for lineage dictating TFs [28]. In this regard, MUC1-C and MYCL were necessary for the expression of (i) NEUROD1, a critical neural TF for Merkel cell development [31–33], and (ii) BRN2, a neural TF and inducer of the NE phenotype [13, 34] (Fig. 4G, left and right). We also found that MUC1-C drives expression of the ATOH1 bHLH TF (Fig. 4G, left and right), which is required for the development of Merkel cells [35], and that MUC1 correlates significantly with ATOH1 expression in MCC tumors (Fig. 4H). Notably, silencing MYCL was associated with upregulation of MUC1-C expression (Supplementary Fig. S5A), indicating that MUC1-C drives MYCL in the absence of an autoinductive feedback loop. In support of the MUC1-C→MYCL pathway, silencing MYCL was also associated with suppression of (i) the OSKM+N factors (Supplementary Fig. S5B) and (ii) NEUROD1 and ATOH1, but not BRN2 (Supplementary Fig. S5C), which is driven by MUC1-C→MYC signaling [13]. These results thus provided support for the involvement of MUC1-C in integrating the expression of pluripotency and lineage dictating TFs in MCC progression.

### MUC1-C attenuates the DNA damage-induced apoptotic response in MCC cells

**Fig. 5:**
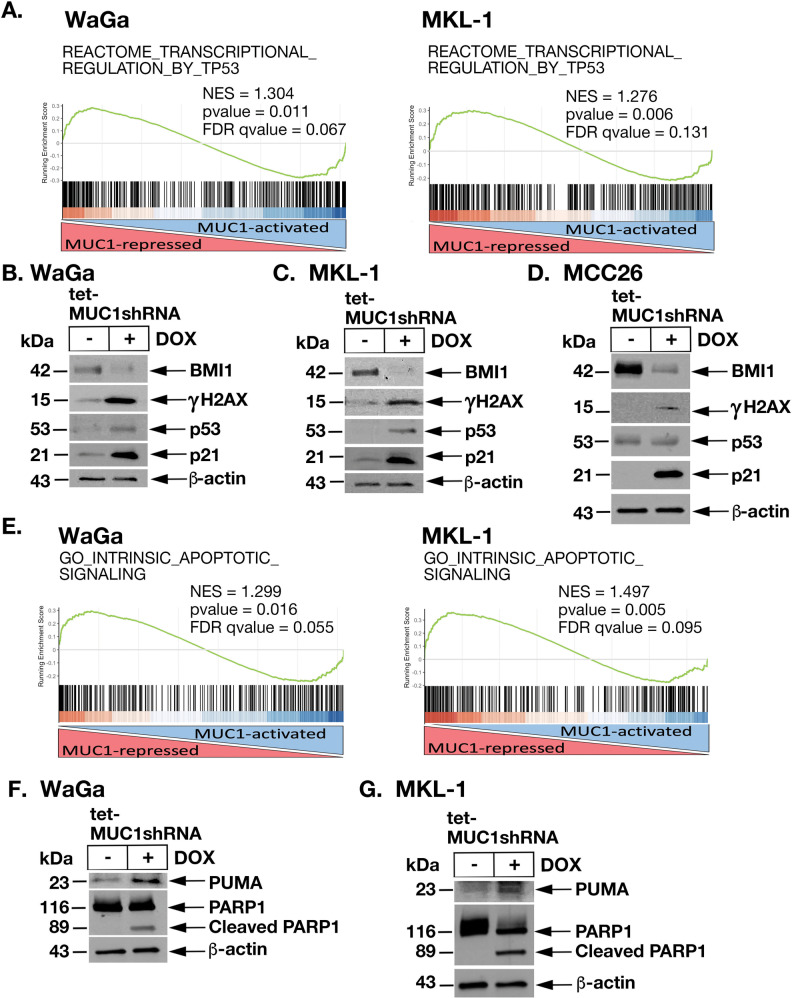
Silencing MUC1-C induces the DNA damage apoptotic response. **A** WaGa (left) and MKL-1 (right) RNA-seq datasets were analyzed with GSEA using the REACTOME TRANSCIPTIONAL REGULATION BY TP53 pathway gene signature. **B**–**D** Lysates from WaGa/tet-MUC1shRNA (**B**), MKL-1/tet-MUC1shRNA (**C**), and MCC26/tet-MUC1shRNA (**D**) cells treated with vehicle or DOX for 3, 5, and 4 days, respectively, were immunoblotted with antibodies against the indicated proteins**. E** WaGa (left) and MKL-1 (right) RNA-seq datasets were analyzed with GSEA using the GO INTRINSIC APOPTOTIC SIGNALING gene signature. **F**, **G** Lysates from WaGa/tet-MUC1shRNA (**F**) and MKL-1/tet-MUC1shRNA (**G**) cells treated with vehicle or DOX for 4 and 6 days, respectively, were immunoblotted with antibodies against the indicated proteins.

WaGa and MKL-1 cells are TP53 wild-type, whereas MCC26 cells are TP53 mutant. In searching for MUC1-regulated gene signatures reflecting this distinction, we found that MUC1-C is significantly associated with activation of the REACTOME TRANSCIPTIONAL REGULATION BY TP53 pathway in WaGa and MKL-1, but not in TP53 mutant MCC26, cells (Fig. 5A, left and right; Supplementary Fig. S6A, left and right). Subsets of upregulated and downregulated TP53 pathway genes common to WaGa and MKL-1 cells included *CDKN1A*, which is activated in the DDR (Supplementary Fig. S6B). MUC1-C interacts directly with p53 in response to DNA replicative stress, promotes the repair of DNA double-strand breaks (DSBs), and suppresses the p53-dependent apoptotic response to DNA damage [8, 16–18]. Mechanistically, MUC1-C integrates induction of BMI1, a component of the polycomb repressive complex 1 (PCR1), with chromatin remodeling and activation of PARP1 in the DNA damage response (DDR) [18, 36]. In accordance with this involvement in the DDR, silencing MUC1-C in WaGa cells suppressed BMI1 expression and induced DSBs, as evidenced by increases in γH2AX (Fig. 5B). Silencing MUC1-C increased p53 levels and expression of the CDK inhibitor p21 (Fig. 5B) [37, 38]. Similar results were obtained in MKL-1 cells (Fig. 5C) and MCC26 cells (Fig. 5D); that is suppression of BMI1 and induction of DNA damage; although in p53 mutant MCC26 cells, there was no detectable increase in p53 levels (Fig. 5D). Notably, the rescue of MUC1-C expression in WaGa/tet-MUC1shRNA cells was associated with recovery of MYCL and BMI1 levels and attenuation of γH2AX upregulation (Supplementary Fig. S6C). Similar results were obtained in MKL-1 cells (Supplementary Fig S6D), indicating that silencing MUC1-C suppresses BMI1 and thereby promotes DNA damage. These results were supported by the finding in MCC tumors that MUC1 expression is significantly associated with activation of the HALLMARK DNA REPAIR gene signature (Supplementary Fig. S6E). MCC cells are highly resistant to genotoxic stress and the induction of apoptosis [1]. Nonetheless, silencing MUC1-C in WaGa and MKL-1 cells was associated with activation of the GO INTRINSIC APOPTOTIC SIGNALING PATHWAY (Fig. 5E, left and right; Supplementary Fig. S6F) and enhanced sensitivity to DNA damage, as evidenced by increased cell death in response to treatment with the genotoxic agent, etoposide (Supplementary Fig. S6G). Moreover, silencing MUC1-C resulted in induction of (i) the p53 downstream PUMA pro-apoptotic effector of mitochondrial outer membrane depolarization [39], (ii) cleavage of PARP1 (Fig. 5F, G), and (iii) apoptosis as determined by annexin V/propidium iodide staining (Supplementary Fig. S7A, B). In addition, p53 mutant MCC26 cells responded to MUC1-C silencing with activation of the GO INTRINSIC APOPTOTIC SIGNALING PATHWAY (Supplementary Fig. S7C) and the induction of apoptosis (Supplementary Fig. S7D), indicating that both MCCP and MCCN cells are addicted to MUC1-C for survival. In further support for the MUC1-C→MYCL pathway, silencing MYCL was also associated with suppression of BMI1 and induction of p21, γH2AX, and PARP1 cleavage (Supplementary Fig. S7E).

### Targeting MUC1-C inhibits MCC self-renewal capacity and tumorigenicity

**Fig. 6:**
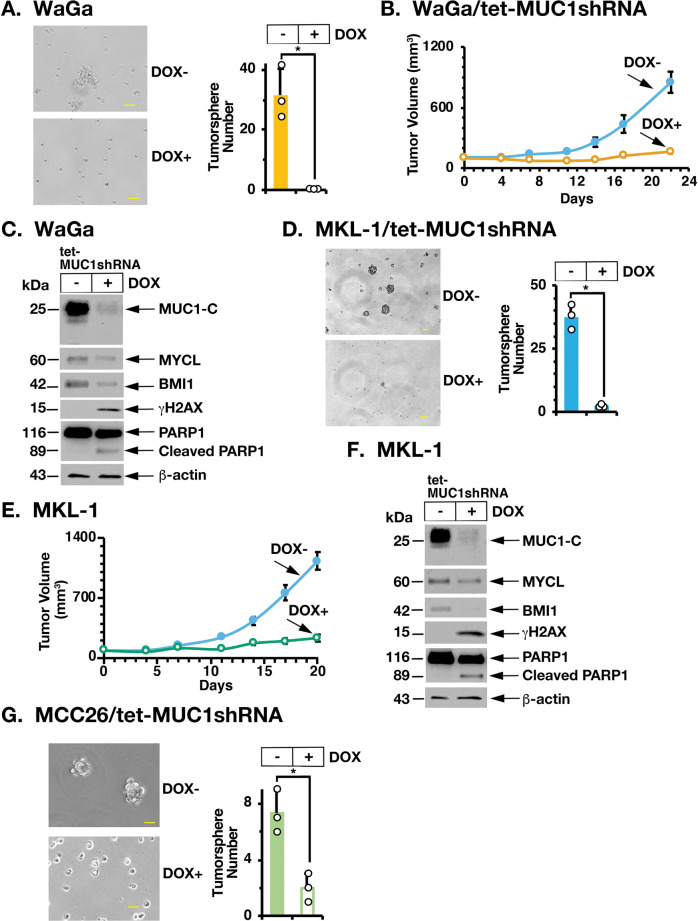
Silencing MUC1-C inhibits MCC self-renewal capacity and tumorigenicity. **A** Representative images of tumorspheres derived from WaGa/tet-MUC1shRNA cells treated with control vehicle or DOX for 7 days (left). Bar represents 50 microns. The number of tumorspheres is expressed as the mean ± SD of three determinations (right). **B**, **C** Six-week-old NSG mice were injected subcutaneously in the flank with 1 × 10^7^ WaGa/tet-MUC1shRNA cells. Mice were pair-matched into two groups when tumors reached 100–150 mm^3^ and were fed without and with DOX. Tumor volumes are expressed as the mean ± SEM for six mice (**B**). Lysates from untreated and DOX-treated WaGa/tet-MUC1shRNA tumors obtained on day 5 were immunoblotted with antibodies against the indicated proteins (**C**). **D** Representative images of tumorspheres derived from MKL-1/tet-MUC1shRNA cells treated with control vehicle or DOX for 7 days (left). Bar represents 50 microns. The number of tumorspheres is expressed as the mean ± SD of three determinations (right). **E**, **F**. Six-week-old NSG mice were injected subcutaneously in the flank with 1 × 10^7^ MKL-1/tet-MUC1shRNA cells. Mice were pair-matched into two groups when tumors reached 100–150 mm^3^ and were fed without and with DOX. Tumor volumes are expressed as the mean ± SEM for six mice (**E**). Lysates from untreated and DOX-treated MKL-1/tet-MUC1shRNA tumors obtained on day 7 were immunoblotted with antibodies against the indicated proteins (**F**). **G** Representative images of tumorspheres derived from MCC26/tet-MUC1shRNA cells treated with control vehicle or DOX for 7 days (left). Bar represents 50 microns. The number of tumorspheres is expressed as the mean ± SD of three determinations (right).

MUC1-C promotes the CSC state by inducing effectors, such as BMI1, that contribute to stemness and self-renewal capacity [8]. In addressing whether MUC1-C drives these hallmark traits in MCC cells, we found that silencing MUC1-C suppresses the formation of WaGa tumorspheres (Fig. 6A, left and right). We also found that rescuing MYCL expression in MUC1-C-silenced WaGa cells (Supplementary Fig. S8A) reverses the suppression of proliferation (Supplementary Fig. S8B) and tumorsphere formation (Supplementary Fig. S8C), in support of the importance of the MUC1-C→MYCL pathway in driving self-renewal capacity. Consistent with these results, the growth of WaGa/tet-MUC1shRNA, but not WaGa/tet-CshRNA, tumor xenografts in NSG mice was inhibited by DOX treatment (Fig. 6B and Supplementary Fig. S9) and was associated with (i) downregulation of MUC1-C, MYCL, and BMI1, (ii) induction of DNA damage, as evidenced by increases in γH2AX, and (iii) apoptotic cell death, as supported by PARP1 cleavage (Fig. 6C). In studies of MKL-1 cells, silencing MUC1-C suppressed tumorsphere formation (Fig. 6D, left and right). Silencing MUC1-C also suppressed MKL-1 tumorigenicity (Fig. 6E) in association with (i) downregulation of MYCL and BMI1 and (ii) induction of γH2AX and PARP1 cleavage (Fig. 6F). In extending these studies to MCC26 cells, we also found that MUC1-C is necessary for tumorsphere formation (Fig. 6G). For a gain-of-function model, we established MCC13 cells, which have low MUC1-C levels, to express a tet-MUC1-C vector (Supplementary Fig. S10A). Treatment of MCC13/tet-MUC1-C cells with DOX increased proliferation (Supplementary Fig. S10B) and tumorsphere formation (Supplementary Fig. S10C), indicating that MUC1-C drives self-renewal of MCCP and MCCN cells.

**Fig. 7:**
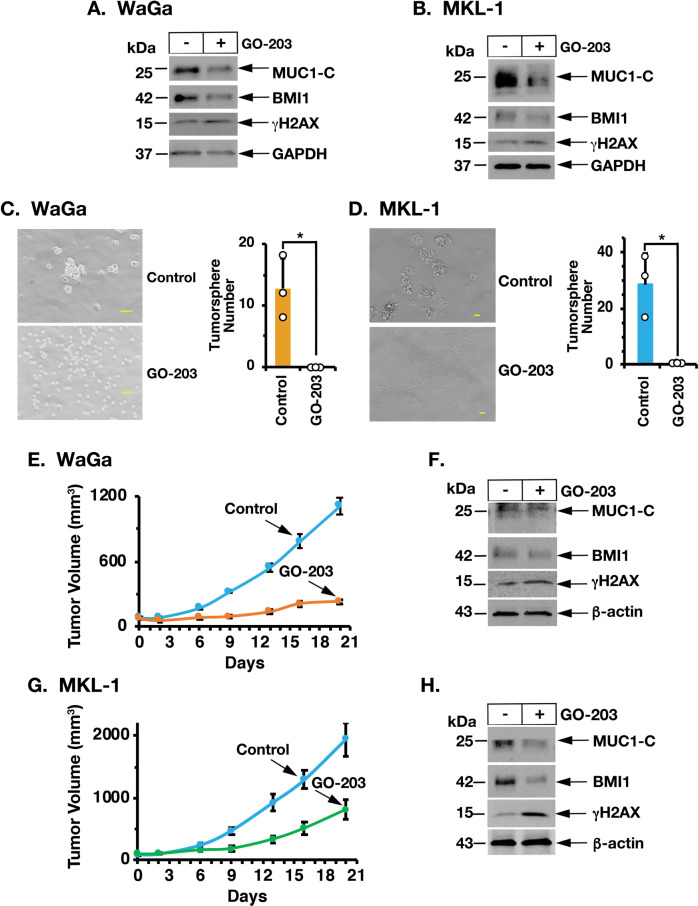
Targeting MUC1-C with the GO-203 inhibitor suppresses MCC self-renewal capacity and tumorigenicity. **A**, **B**. Lysates from WaGa (**A**) and MKL-1 (**B**) cells treated with 0.15 and 0.25 μM GO-203 for 24 h were immunoblotted with antibodies against the indicated proteins. **C**, **D** Representative images of tumorspheres derived from WaGa (**C**) and MKL-1 (**D**) cells treated with 5 μM GO-203 for 7 days (left). Bar represents 50 microns. The number of tumorspheres is expressed as the mean ± SD of three determinations (right). **E**, **F**. Six-week-old NSG mice were injected subcutaneously in the flank with 1 × 10^7^ WaGa cells. Mice pair-matched into two groups when tumors reached 100–150 mm^3^ were treated intraperitoneally each day with PBS or GO-203. Tumor volumes are expressed as the mean ± SEM for six mice (**E**). Lysates from tumors harvested on days 20–21 were immunoblotted with antibodies against the indicated proteins (**F**). **G**, **H** Six-week-old NSG mice were injected subcutaneously in the flank with 1 × 10^7^ MKL-1 cells. Mice pair-matched into two groups when tumors reached 100–150 mm^3^ were treated intraperitoneally each day with PBS or GO-203. Tumor volumes are expressed as the mean ± SEM for six mice (**G**). Lysates from tumors harvested on days 20–21 were immunoblotted with antibodies against the indicated proteins (**H**).

The findings that MUC1-C drives MCC cell self-renewal capacity hold potentially important implications for MCC treatment. In this respect, we investigated the effects of the GO-203 inhibitor, which blocks MUC1-C homodimerization and phenocopies the effects of MUC1-C silencing [8]. Along these lines, treatment of WaGa cells with GO-203 in vitro was associated with (i) suppression of BMI1 and induction of γH2AX (Fig. 7A), (ii) pronounced inhibitory effects on growth (Supplementary Fig. S11A), and (iii) induction of apoptosis (Supplementary Fig. S11B). Similar results were obtained when treating MKL-1 cells with GO-203 (Fig. 7B and Supplementary Fig. S11C, D). GO-203 was also effective in inhibiting WaGa (Fig. 7C, left and right) and MKL-1 (Fig. 7D, left and right) tumorsphere formation. Moreover, targeting MUC1-C with GO-203 was effective in inhibiting WaGa (Fig. 7E, F) and MKL-1 (Fig. 7G, H) tumorigenicity in association with suppressing BMI1 and inducing DNA damage.

## Discussion

Treatment of MCC has historically been limited by the emergence of resistance to genotoxic anti-cancer agents [1, 2]. This aside, the effectiveness of treating MCC with ICIs has markedly changed the management of unresectable and metastatic disease [40]. These seminal advances notwithstanding, there remain no recognized targets that contribute to MCC progression and are druggable. The present work demonstrates that *MUC1* is widely expressed in MCC tissues and at higher levels in MCCP, relative to MCCN, tumors. Studies in classical WaGa and MKL-1 MCCP cells, which closely share transcriptomes with MCC tumors [22], demonstrated higher levels of MUC1-C expression compared to that in variant MCC13, MCC26, and UISO MCCN lines. These results invoked the possibility that MCPyV increases MUC1-C levels in MCCP cells. Indeed, we found a significant correlation between MCPyV and MUC1 expression in MCC tumors. However, there was no apparent effect of silencing the MCPyV LT and ST antigens on MUC1-C levels in MKL-1 cells. Moreover, silencing MUC1-C in WaGa and MKL-1 cells had little if any effect on the expression of the LT and ST antigens. Additional investigation will therefore be needed to define the mechanistic basis for the intriguing upregulation of MUC1-C in MCCP tumors and cells. Along these lines, *MUC1* evolved in mammals to protect barrier epithelia, such as skin, from loss of homeostasis [8]. *MUC1* is induced in response to viral infections, which may be related to the activation of cytosolic nucleotide receptors by the presence of viral DNA and contribute to increased levels of MUC1-C expression in MCCP cells [8]. Nonetheless, we conclude at this point that levels of MUC1-C expression in MCC cells are apparently unrelated to the extent of their addiction. In that sense, we found that WaGa and MKL-1 cells are highly dependent on MUC1-C for their survival and that remarkably similar results were obtained in MCC26 MCCN cells, which have much lower levels of MUC1-C expression. Noteworthy are the observations that classical MCCP cell lines express wild-type p53 and RB, in contrast to their variant MCCN counterparts. MUC1-C expression is linked to suppression of p53 and RB and thus could downregulate these pathways in MCCP cells [8].

MCC tumors and cell lines overexpress MYCL [4]. In addition, MCCP cells are dependent on MYCL for survival [4]. In promoting this dependence, binding of MCPyV ST to MYCL recruits the EP400 histone acetyltransferase and chromatin remodeling complex in driving MCCP gene expression [4]. We found that silencing MUC1-C in MCCP and MCCN cells results in the partial downregulation of MYCL expression and that rescue of MUC1-C downregulation restores MYCL levels. We also found that MUC1-C forms a nuclear complex with MYCL and that MUC1-C binds directly to MYCL. MUC1-C binds directly to the MYC HLH-LZ domain and promotes induction MYC target genes [27]. Our results indicate that MUC1-C is necessary for the expression of MYCL transcripts and protein, which could be regulated by transcriptional and/or miRNA-mediated, as well as posttranslational, mechanisms. Accordingly, additional studies will be needed to determine if MUC1-C activates the MYCL pathway by regulating MYCL expression and the MYCL transactivation function. In support of the potential importance of a MUC1-C→MYCL pathway, we found that MUC1-C and MYCL regulate common sets of gene signatures that include in part pluripotency and NE lineage dictating TFs. In this context, MUC1-C was necessary for driving the expression of the OSKM+NANOG pluripotency factors. These findings were of interest in that (i) MYC is also upregulated in MCC cells, (ii) MCPyV ST antigen stabilizes MYC and in combination with OSK induces pluripotent stem cells and (iii) silencing MYC inhibits MCC cell growth [41]. Accordingly, MUC1-C could promote MCC progression by activating MYCL and/or MYC, depending on cell context or MCPyV status. Induction of pluripotency factors contributes to lineage plasticity in driving NE dedifferentiation [13]. Consistent with this capacity in MCC cells, MUC1-C was necessary for the expression of the NE lineage dictating NEUROD1, BRN2, and ATOH1 TFs. Lineage plasticity and, significantly, NE differentiation in cancer cells is also associated with activation of the replication stress response, which is dependent on DNA repair and cell cycle checkpoints for proliferation [42–44]. MCC tumors acquire resistance to treatment with genotoxic anti-cancer agents, albeit by unknown mechanisms, that are associated with lack of response and recurrence of disease [1]. Our results may hold potential therapeutic implications in this regard in that silencing MUC1-C in MCCP and MCCN cells was associated with the rapid induction of DNA replication stress, indicating that this response is independent of MCPyV status.

MUC1-C has been linked to DNA damage resistance by promoting ATM- and PARP1-mediated repair of DSBs [17, 18]. MUC1-C signaling further contributes to the DDR by MYC-mediated activation of BMI1 [11, 36], which associates with MUC1-C/PARP1 complexes and facilitates DSB repair by inducing H2A ubiquitylation [18]. Silencing MUC1-C in MCCP cells suppresses BMI1 expression in association with the accumulation of DSBs and, as a result, activation of the p53 response. In further support for this MUC1-C driven pathway of DNA damage resistance, silencing MUC1-C in WaGa and MKL-1 cells activated the REACTOME TRANSCRIPTIONAL REGULATION BY TP53 pathway with the induction of effectors, such as p21 and PUMA, that govern cell cycle arrest and apoptotic cell death [38, 45]. Adding to this complexity of MUC1-C addiction, mutant p53 MCC26 cells also responded to MUC1-C silencing with induction of DNA damage, inhibition of growth, and induction of death. In this setting, addiction to MUC1-C loss occurred in the absence of activating p53 target genes and inducing PUMA; whereas silencing MUC1-C in WaGa and MKL-1 cells induced the GO INTRINSIC APOPTOTIC SIGNALING PATHWAY gene signature and apoptotic cell death. Despite these different pathways, our results demonstrate that targeting MUC1-C in MCCP and MCCN cells results in similar outcomes with loss of survival, in support of oncogene addiction [20]. By extension, the findings that targeting MUC1-C in MCCP and MCCN cells induces DNA damage lends support for designing potential strategies to improve MCC treatment regardless of MCPyV status. Increasing evidence indicates that the MUC1-C is necessary for the self-renewal of CSCs [8]. In support of that notion, MUC1-C drives the progression of NE prostate CSCs at least in part by remodeling the chromatin architecture [13, 14, 25, 46]. Along these lines, agents targeting MUC1-C, such as CAR T cells, antibody-drug conjugates, and GO-203, in preclinical and clinical development [8] could be effective alone and in combination for the treatment of MCCP and MCCN tumors.

## Materials and methods

### Analysis of human MCC tumor datasets

Data analysis was performed using the cBioPortal Cancer Genomic and Oncomine websites [47, 48].

### Immunohistochemistry (IHC) staining

Formalin-fixed, paraffin-embedded sections of MCC tissue samples were deparaffinized in xylene, graded concentrations of EtOH and then distilled water. Antigen retrieval was performed in EDTA buffer, pH 8.5 (E1161, Sigma-Aldrich, Saint Louis, MO, USA). Peroxidase blocking was performed with Peroxidazed 1 (Biocare Medical, Pacheo, CA, USA) for 5 min and then Background Sniper (Biocare Medical) for 10 min. Slides were incubated with anti-MUC1-C (dilution 1:100, MA5-11202; Thermo Fisher Scientific) for 2 h, anti-Armenian hamster secondary antibody (dilution 1:200, ab5745; Abcam, Cambridge, MA, USA) for 30 min and diaminobenzidine tetrahydrochloride chromagen reagents for 5 min at room temperature. Immunostained sections were counterstained with hematoxylin.

### Cell culture

WaGa, MKL-1, MCC13, MCC26, and UISO MCC cells were obtained as described [4, 49] and cultured in RPMI 1640 medium (Corning, Corning, NY, USA) supplemented with 10% FBS and 2 mM glutamine. Cells were cultured for 3-4 months. Authentication of the cells was performed by short tandem repeat analysis. Cells were monitored for mycoplasma contamination using the MycoAlert Mycoplasma Detection Kit (Lonza, Rockland, ME, USA).

### Gene silencing and rescue

MUC1shRNA (MISSION shRNA TRCN0000122938; Sigma) or a control scrambled shRNA (CshRNA; Sigma) was inserted into the pLKO-tet-puro vector (Plasmid #21915; Addgene, Cambridge, MA, USA) as described [13]. MYCLshRNA was inserted into pLKO-tet-puro vector as described [4]. MUC1-C or Flag-tagged MUC1-CD [17] was inserted into pInducer20 (Plasmid #44012, Addgene) [50]. MYCL [4] was inserted into the empty control pLenti CMV Blast DEST (706-1) vector (Plasmid #17451, Addgene). Cells transduced with the vectors were selected for growth in 1–4 μg/ml puromycin, 400–1000 μg/ml hygromycin, or 10 μg/ml blasticidin. Cells were (i) treated with 0.1% DMSO as the vehicle control or 500 ng/ml DOX (Millipore Sigma) and (ii) transfected with a MUC1/ASO (LG00788741; Qiagen, Hilden, Germany) or a control C/ASO (LG00000001; Qiagen) in the presence of Lipofectamine 3000 Reagent (Thermo Fisher Scientific, Waltham, MA, USA).

### Quantitative reverse-transcription PCR (qRT-PCR)

Total cellular RNA was isolated using Trizol reagent (Thermo Fisher Scientific). cDNAs were synthesized using the High Capacity cDNA Reverse Transcription Kit (Applied Biosystems, Grand Island, NY, USA). The cDNA samples were amplified using the Power SYBR Green PCR Master Mix (Applied Biosystems) and the CFX96 Real-Time PCR System (BIO-RAD, Hercules, CA, USA) as described [13]. Primers used for qRT-PCR are listed in Supplementary Table S1.

### Immunoblot analysis

Total lysates prepared from subconfluent cells were subjected to immunoblot analysis using anti-MUC1-C (HM-1630-P1ABX, 1:1000 dilution; Thermo Fisher Scientific), anti-GAPDH (5174, 1:1000 dilution; Cell Signaling Technology), anti-β-actin (A5441, 1:5000 dilution; Sigma-Aldrich), anti- γH2AX (9718, 1:1000 dilution; CST), anti-BMI1 (6964, 1:2000 dilution; CST), anti-MYCL (PA5-109998, 1:1000 dilution; Invitrogen, Waltham, MA), anti-MYC (ab32072, 1:1000 dilution; Abcam), anti-SOX2 (3579, 1:1000 dilution; CST), anti-KLF4 (12173, 1:1000 dilution; CST), anti-OCT4 (2750, 1:1000 dilution; CST), anti-NEUROD1 (4373, 1:1000 dilution; CST), anti-BRN2 (12137, 1:1000 dilution; CST), anti-ATOH1 (21215-1-AP, 1:2000 dilution; Proteintech, Rosemont, IL, USA), anti-PARP1 (9532, 1:1000 dilution; CST), anti-NANOG (4903, 1:2000 dilution; CST), anti-p53 (9282, 1:1000 dilution; CST), anti-p21 (2947, 1:1000 dilution; CST), and anti-PUMA (12450, 1:1000 dilution; CST).

### Coimmunoprecipitation of nuclear proteins

Nuclear lysates were isolated as described [14]. DNA was digested by incubation in 20 U/ml DNase for 30 min at 37 °C. Nuclear proteins were incubated with anti-MUC1-C (#MA5-11202; Thermo Fisher Scientific) at 4 °C overnight and then precipitated with Dynabeads Protein G (10003D; Thermo Fisher Scientific) for 2 h at 4 °C. Beads were washed twice with washing buffer (20 mM Tris-HCl, pH 8.0, 0.2 mM EDTA, 1.5 mM MgCl_2_, 0.5% NP40, and 150 mM NaCl) and once with 10% TE buffer (BM-304A; Boston BioProducts), and then resuspended in sample loading buffer.

### Direct binding studies

EF1a_MYCL_P2A_Hygro_Barcode (Plasmid #120462, Addgene) was used to construct and purify full-length (FL) GST-MYCL (aa 1–364). GST-MYCL protein was cleaved with thrombin to remove GST. GST and GST-MUC1-CD (FL; aa 1–72) were prepared as described [27]. Equimolar amounts of purified MYCL were incubated with GST or GST-MUC1-CD proteins bound to glutathione beads, and the adsorbates were analyzed by immunoblotting with anti-MYCL.

### Apoptosis assays

Cells were harvested and stained with Annexin V Alexa Fluor 488 and propidium iodide using the Dead Cell Apoptosis Kit (V13241; Thermo Scientific, Rockford, IL, USA). The cell apoptosis ratio was measured according to the manufacturer’s instructions by flow cytometry.

### RNA-seq analysis

Total RNA from cells cultured in triplicates was isolated using the RNeasy Plus Mini Kit (Qiagen). TruSeq Stranded mRNA (Illumina, San Diego, CA, USA) was used for library preparation as described [13]. Raw sequencing reads were aligned to the human genome (GRCh38.74) using STAR. Raw feature counts were normalized and differential expression analysis using DESeq2. Differential expression rank order was utilized for subsequent GSEA, performed using the fgsea (v1.8.0) package in R. Gene sets queried included those available through the Molecular Signatures Database (MSigDB).

### Mouse tumor model studies

Six- to eight-week-old NSG mice (Taconic Farms, Germantown, NY, USA) were injected subcutaneously in the flank with 1 × 10^7^ WaGa or MKL-1 cells in 100 μl of a 1:1 solution of medium and Matrigel (BD Biosciences). When the mean tumor volume reached 100–150 mm^3^, mice were pair-matched into groups. In studies of (i) WaGa/tet-CshRNA, WaGa/tet-MUC1shRNA, and MLK-1/tet-MUC1shRNA tumors, mice were fed without or with DOX (625 ppm, daily), and (ii) WaGa and MKL-1 tumors, mice were treated intraperitoneally each day with PBS or GO-203 at a dose of 12 μg/g body weight. In other studies, 10, 5, and 2.5 × 10^6^ WaGa/tet-CshRNA and WaGa/tet-MUC1shRNA cells were implanted into the left and right flanks, respectively, of NSG mice. Unblinded tumor measurements and body weights were recorded twice each week. Mice were sacrificed when tumors reached >2000 mm^3^ as calculated by the formula: (width)^2^ × length/2. These studies were conducted in accordance with ethical regulations required for approval by the Dana-Farber Cancer Institute Animal Care and Use Committee under protocol 03-029.

### Tumorsphere formation assays

Cells (1–3 × 10^4^) were seeded per well in 6-well ultra-low attachment culture plates (Corning Life Sciences) in DMEM/F12 50/50 medium (Corning Life Sciences) with 20 ng/ml EGF (Millipore Sigma), 20 ng/ml bFGF (Millipore Sigma) and 1% B27 supplement (Gibco). In certain studies, cells were (i) treated with vehicle or 500 ng/ml DOX, and (ii) left untreated or treated with GO-203. Tumorspheres were counted under an inverted microscope in triplicate wells.

### Statistical analysis

Each experiment was performed at least three times. Data are expressed as the mean ± SD. The unpaired Mann–Whitney *U* test was used to determine differences between means of groups. A *p* value of <0.05 denoted by an asterisk (*) was considered statistically significant. The “resource equation” method was used to calculate the required number of animals. The formula is “*E* = Total number of animals – Total number of groups”. *E* is the degree of freedom of analysis of variance. The value of *E* between 10 and 20 is considered to be adequate. For each study, the total number of animals is 12, and the total number of groups is 2 with an *E* value of 10 (*E* = 12 – 2 = 10). The number of mice we used is the minimum to be significant.

## Supplementary information

Supplementary Material

## Data Availability

The accession numbers for the RNA-seq data are GEO Submission GSE69878, GSE180876, GSE180890, and GSE180891.
