# Supplementary material for: Addiction of Merkel cell carcinoma to MUC1-C identifies a potential new target for treatment
Source: Oncogene. 2022 Jun 10;41(27):3511–23. doi: 10.1038/s41388-022-02361-3 (PMC9249628; doi:10.1038/s41388-022-02361-3)
Supplement: Supplementary file 1 — Supplementary Material [file 41388_2022_2361_MOESM1_ESM.pdf]

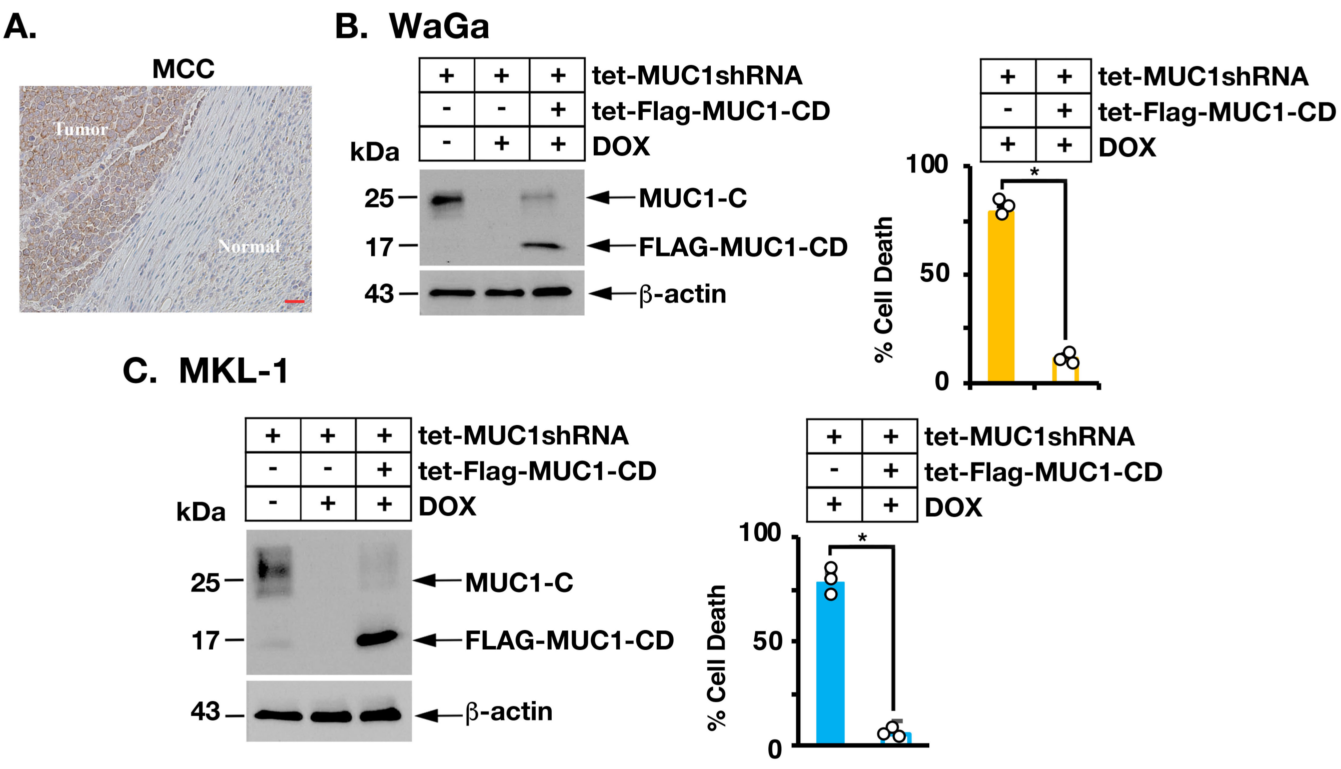

Supplemental Figure S1. Expression of MUC1-C in MCC cells and rescue of MUC1-C silencing with Flag-MUC1-CD. A. IHC staining of MUC1-C in MCCN tumor cells and not surrounding tissue. **B and C.** WaGa (**B**) and MKL-1 (**C**) cells expressing tet-MUC1shRNA and tet-Flag-MUC1-CD were treated with vehicle or DOX for 3 days. Lysates were immunoblotted with antibodies against the indicated proteins (left). Cell death was monitored by trypan blue staining (right). The results are expressed as the % cell death (mean±SD of three separate determinations).

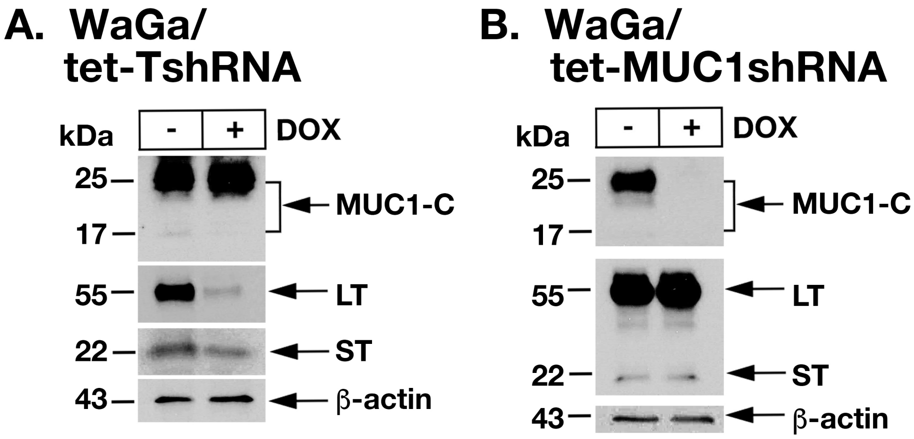

Supplemental Figure S2. Effects of silencing MCPyV LT/ST on MUC1-C expression. A. Lysates from WaGa/tet-MUC1shRNA cells treated with vehicle of DOX for 3 days were immunoblotted with antibodies against the indicated proteins. **B.** Lysates from WaGa/tet-TshRNA cells treated with vehicle or DOX for 3 days were immunoblotted with antibodies against the indicated proteins.

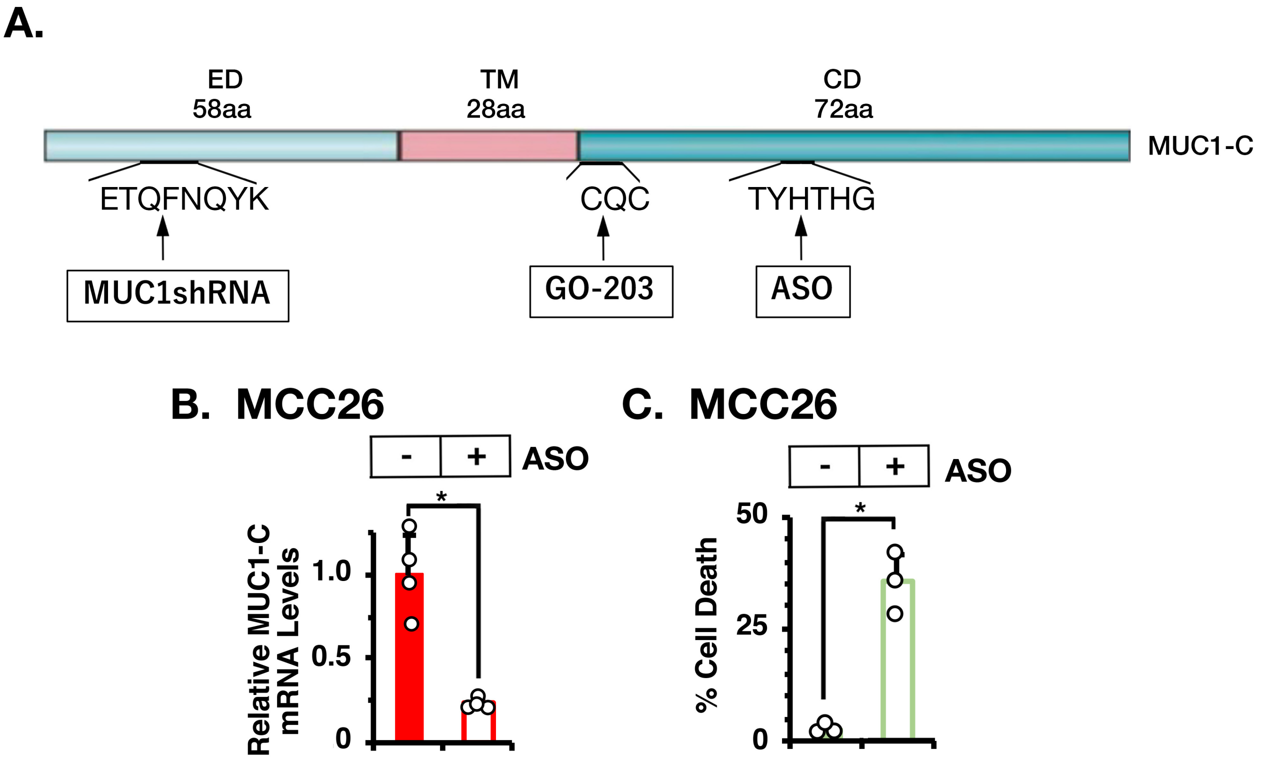

Supplemental Figure S3. Targeting the MUC1-C cytoplasmic domain inhibits MCC26 cell survival. A. Schema of MUC1-C with the 58 aa extracellular domain (ED), 28 aa transmembrane region ™ and the 72 aa cytoplasmic domain. Highlighted are the aa sequences targeted by the MUC1shRNA, the GO-203 inhibitor and the MUC1/ASO. **B.** Lysates from MCC26 cells transfected with 30 nM MUC1/ASO or a control C/ASO for 24 hours were analyzed for MUC1-C mRNA levels by qRT-PCR. The results (mean±SD of three determinations) are expressed as relative mRNA levels compared to that obtained for C/ASO transfected cells (assigned a value of 1). **C.** MCC26 cells transfected with 30 nM MUC1/ASO or C/ASO for 72 hours were monitored for cell death by trypan blue staining. The results are expressed as the % cell death (mean±SD of three separate determinations).

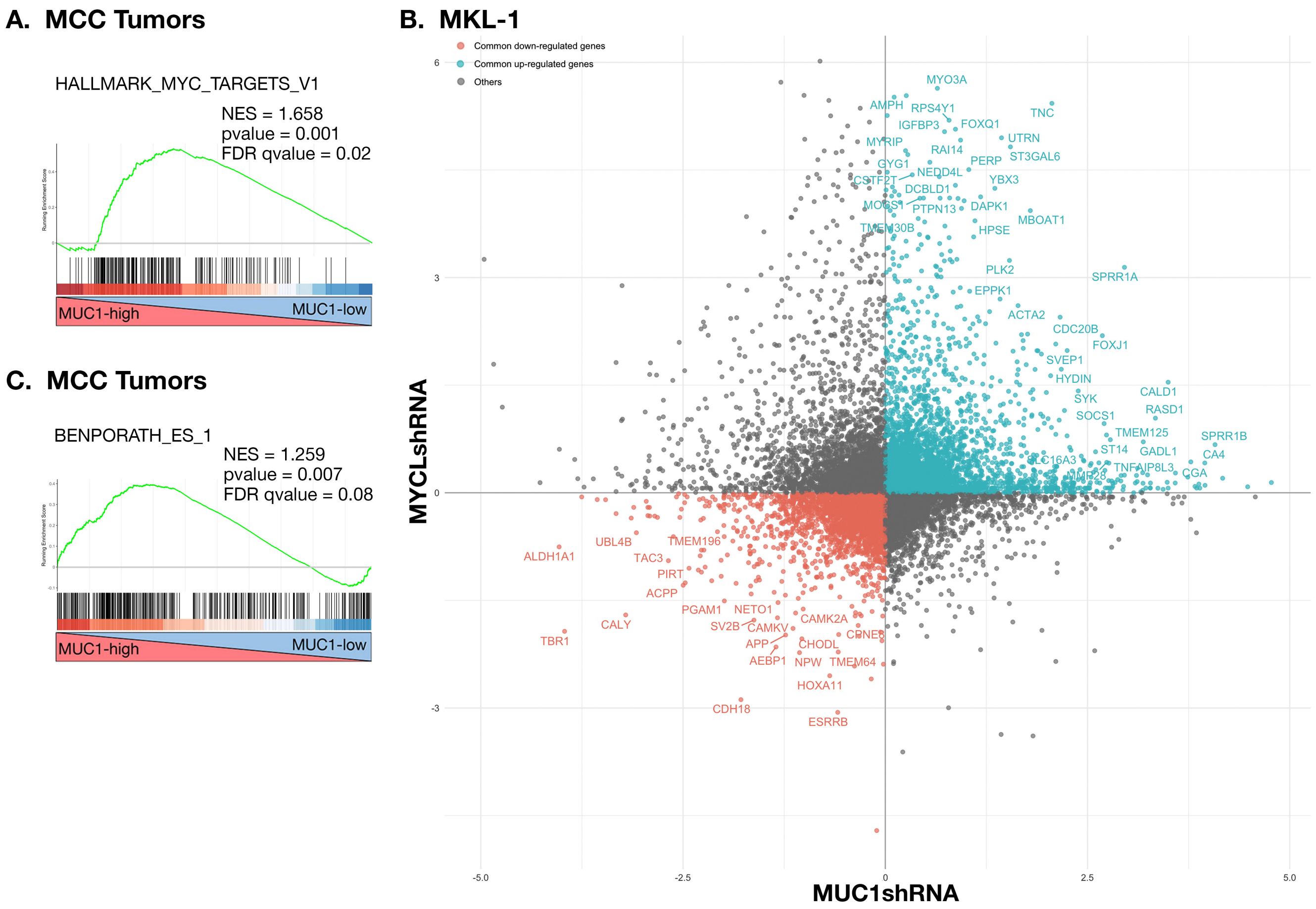

Supplemental Figure S4. Association of MUC1 with activation of MYC target and stemness-associated genes. A. GSEA of the RNA-seq dataset derived from 55 MCCP and MCCN tumors using the HALLMARK MYC TARGETS V1 gene signature. **B.** Overlap of MUC1-C and MYCL regulated genes in MKL-1 cells. **C.** GSEA of the RNA-seq dataset derived from 55 MCCP and MCCN tumors using the BENPORATH ES 1 gene signature.

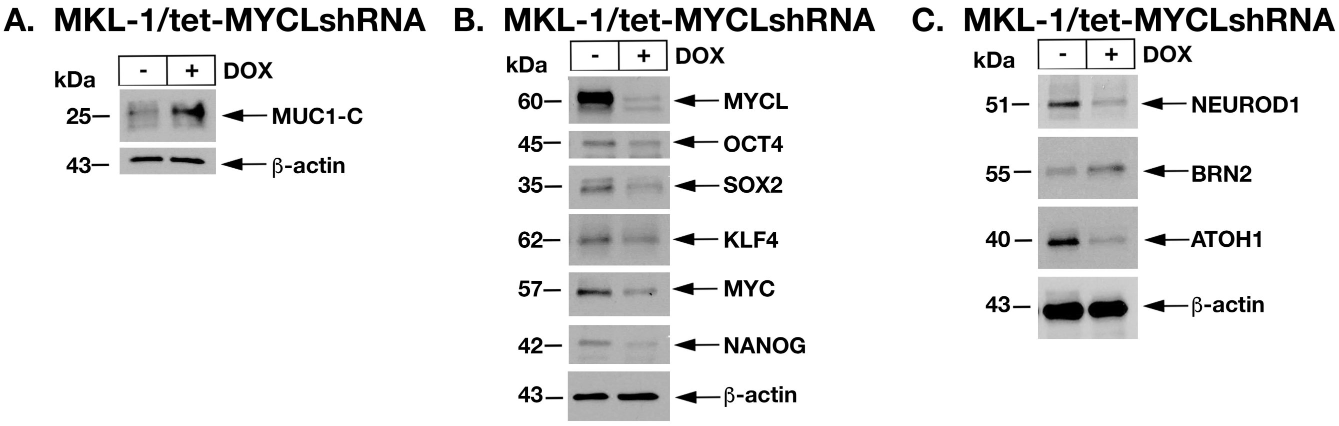

Supplemental Figure S5. Silencing MYCL suppresses OSKM and NE differentiation factor expression in MKL-1 cells. A-C. Lysates from MKL-1/tet-MYCLshRNA cells treated with vehicle or DOX for 6 days were immunoblotted with antibodies against the indicated proteins.

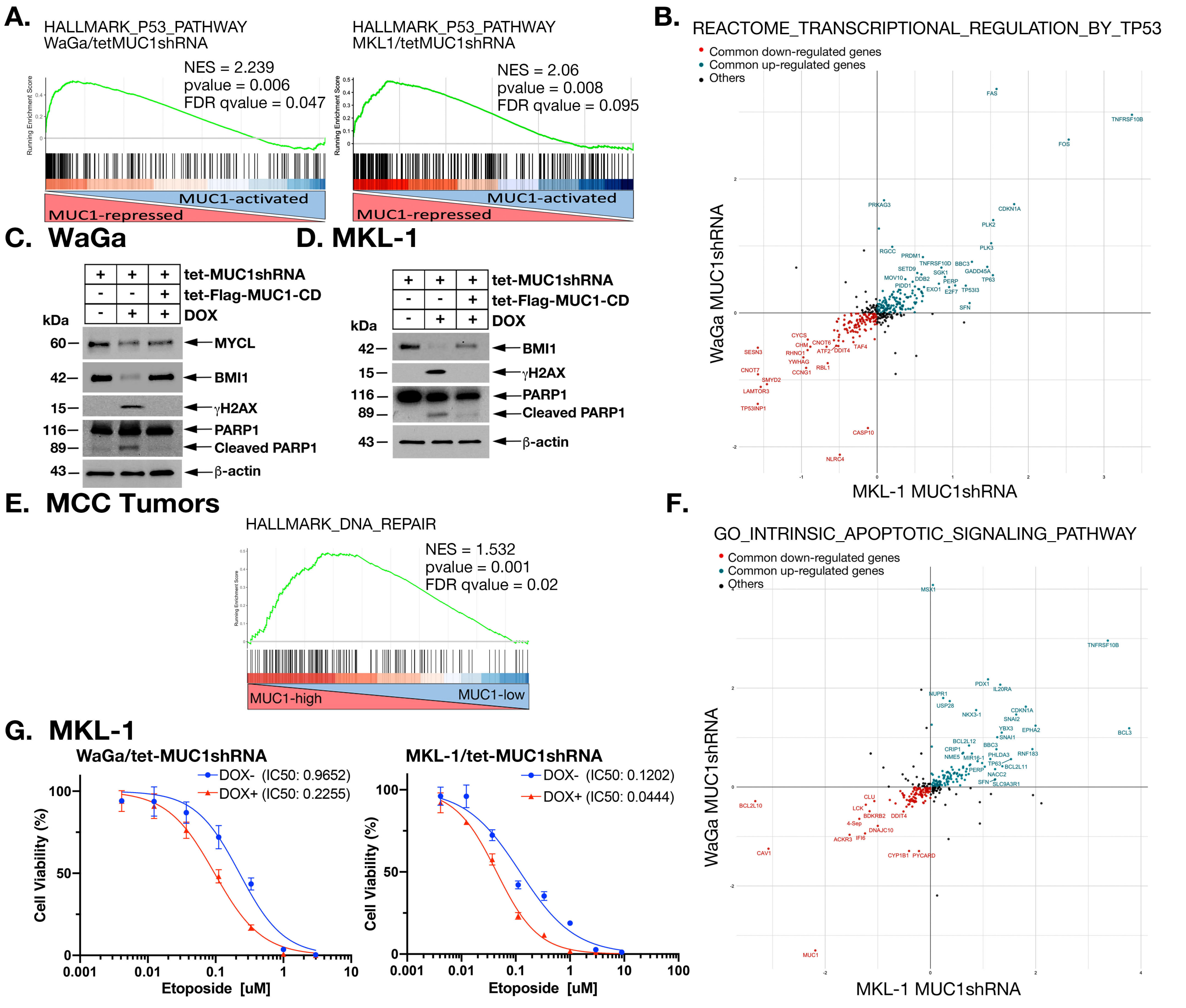

Supplemental Figure S6. MUC1-C regulates TP53 and apoptotic pathway signatures. A. WaGa (left) and MKL-1 (right) RNA-seq datasets were analyzed with GSEA using the HALLMARK P53 pathway gene signature. **B.** Overlap of MUC1-C regulated genes in WaGa and MKL-1 cells using the REACTOME TRANSCRIPTIONAL REGULATION BY TP53 gene signatures. **C and D.** WaGa © and MKL-1 (**D**) cells expressing tet-MUC1shRNA and tet-Flag-MUC1-CD were treated with vehicle or DOX for 3 days. Lysates were immunoblotted with antibodies against the indicated proteins. **E.** GSEA of the RNA-seq dataset derived from 55 MCCP and MCCN tumors using the HALLMARK DNA REPAIR gene signature. **F.** Overlap of MUC1-C regulated genes in WaGa and MKL-1 cells using the GO INTRINSIC APOPTOTIC SIGNALING gene signature. **G.** WaGa/tet-MUC1shRNA (left) and MKL-1/tet-MUC1shRNA (right) cells were treated with vehicle or DOX in the presence of the indicated etoposide concentrations for 72 h. Cell viability was analyzed by Alamar blue staining. The results are expressed as the % cell death (mean±SD of six separate determinations).

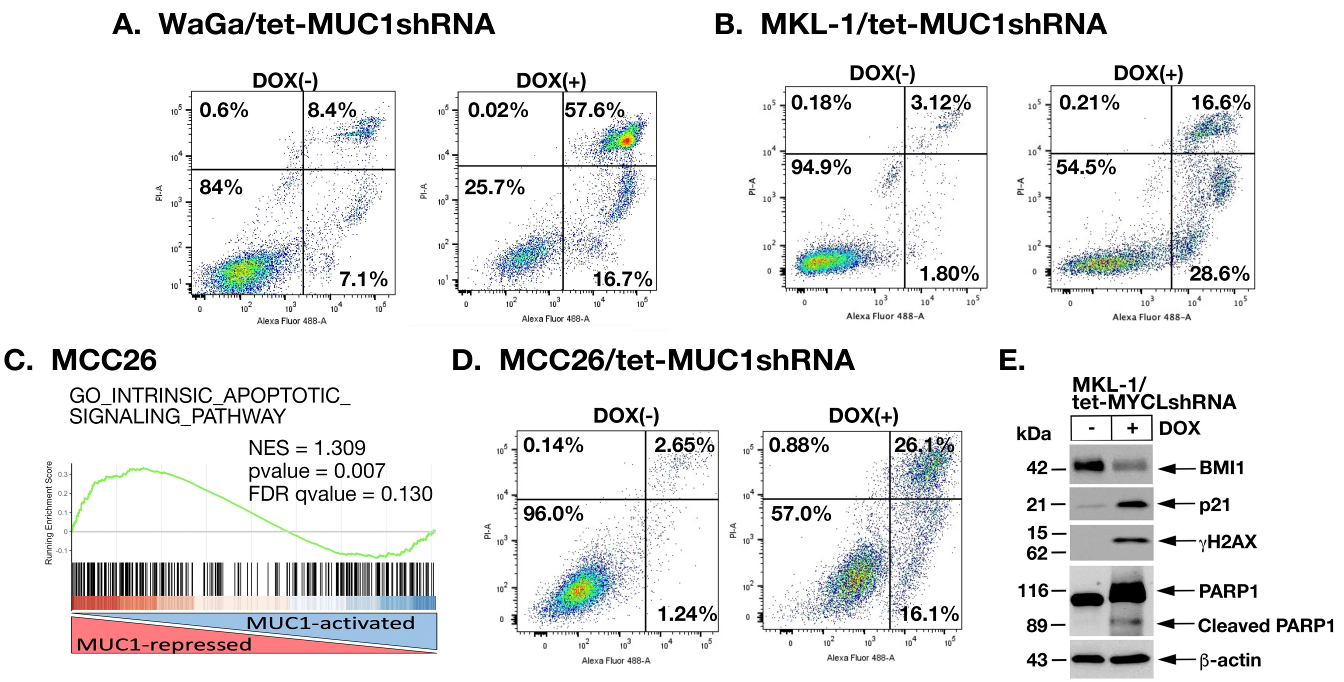

Supplemental Figure S7. Silencing MUC1-C induces apoptosis in MCC cells. A and B. WaGa/tet-MUC1shRNA (**A**) and MKL-1/tet-MUC1shRNA (**B**) cells treated with vehicle or DOX for 4 and 8 days, respectively, were analyzed for PI and annexin V staining by flow cytometry. The results are representative of 3 separate experiments. **C.** MCC26 RNA-seq datasets were analyzed with GSEA using the GO INTRINSIC APOPTOTIC SIGNALING gene signature. **D.** MCC26/tet-MUC1shRNA cells treated with vehicle or DOX for 6 days were analyzed for PI and annexin V staining by flow cytometry. The results are representative of 3 separate experiments. **E.** Lysates from MKL-1/tet-MYCLshRNA cells treated with vehicle or DOX for 6 days were immunoblotted with antibodies against the indicated proteins.

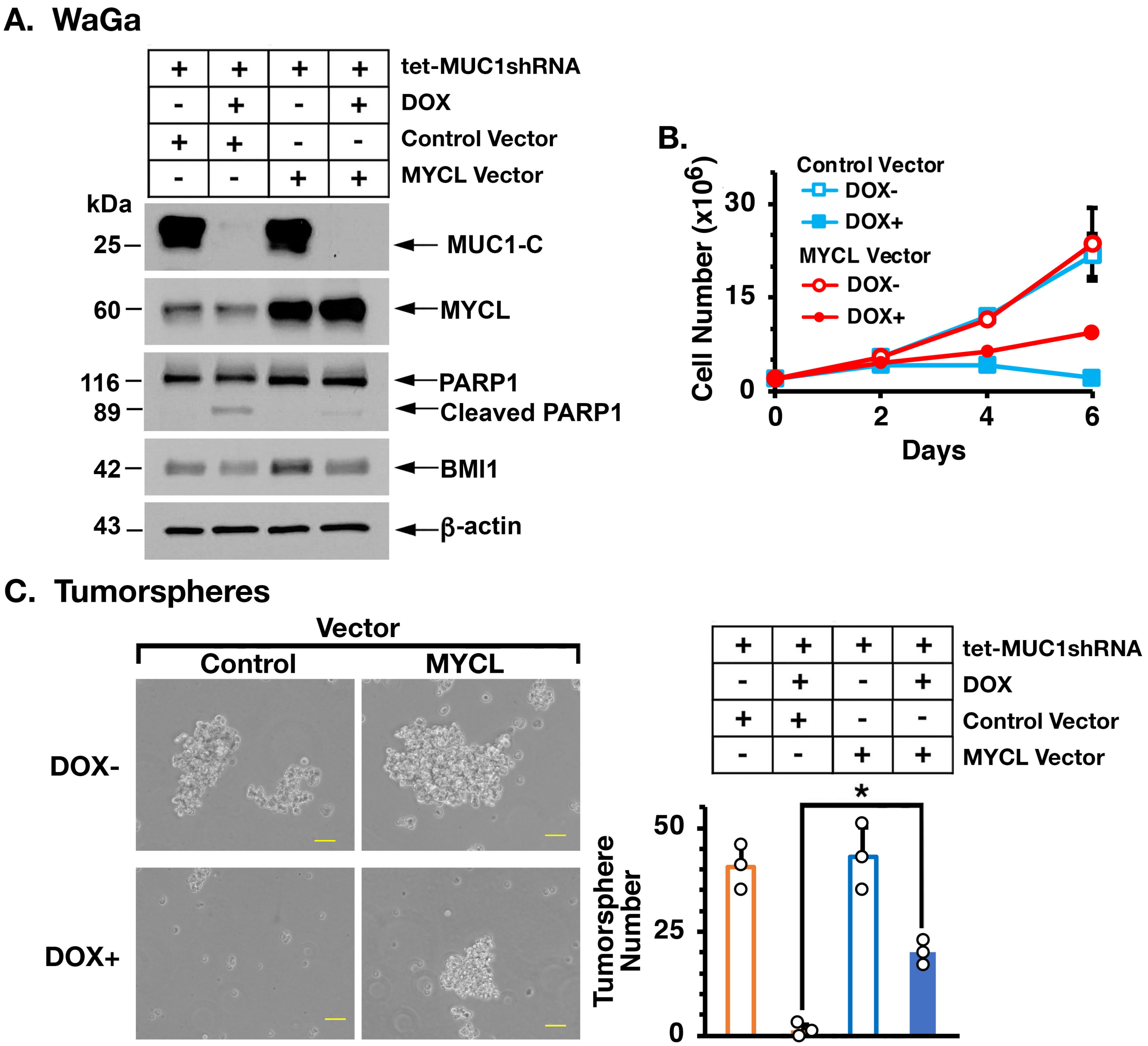

Supplemental Figure S8. Rescue of MYCL in WaGa cells. A-C. WaGa cells expressing tet-MUC1shRNA and tet-MYCL were treated with vehicle or DOX for 4 (**A**), 6 (**B**) and 7 (**C**) days. **A.** Lysates were immunoblotted with antibodies against the indicated proteins. **B.** Cells were analyzed for proliferation by trypan blue staining. The results are expressed as the mean±SD of three separate determinations. **C.** Representative images of tumorspheres (left). Bar represents 50 microns. The number of tumorspheres is expressed as the mean±SD of three determinations (right).

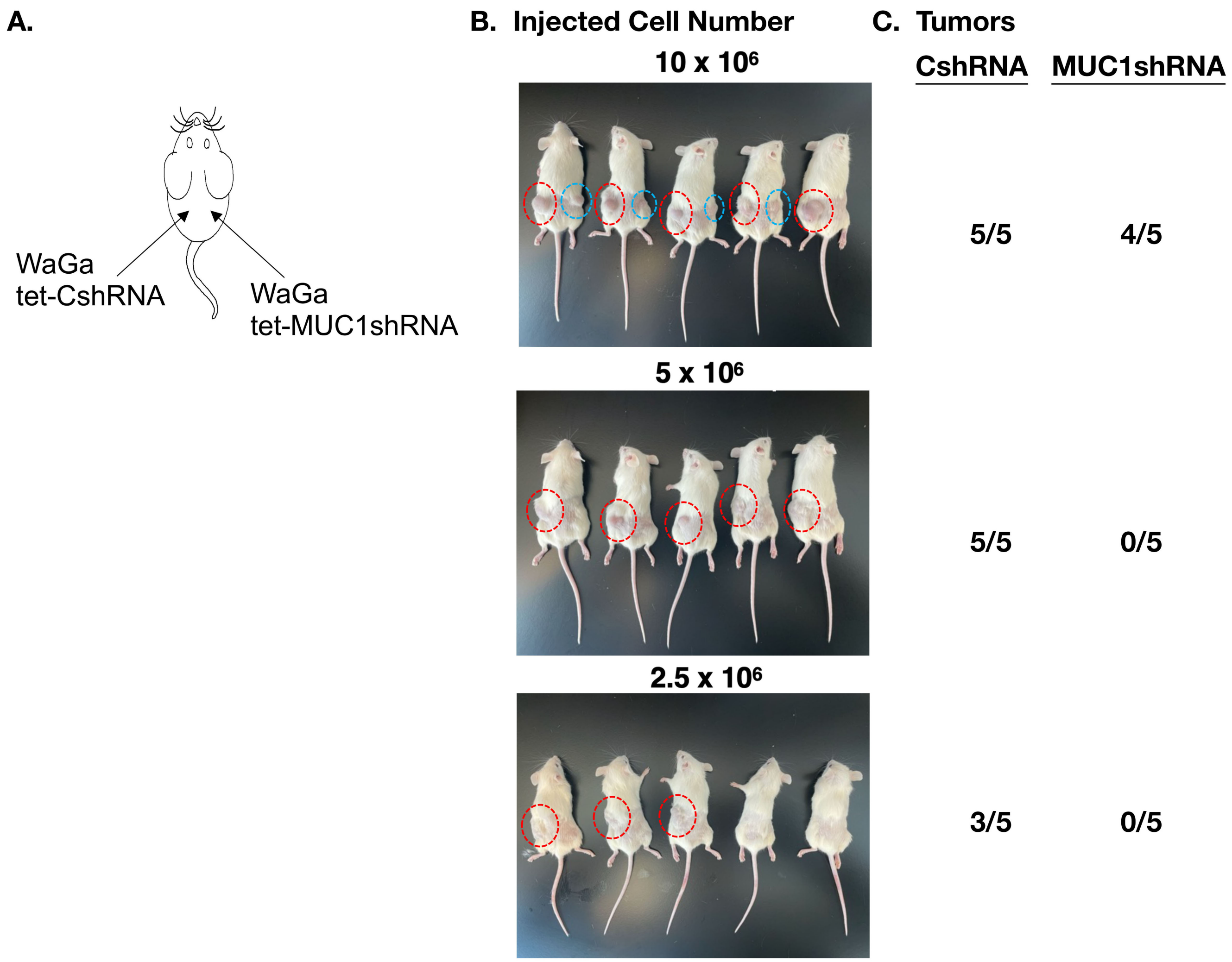

Supplemental Figure S9. *In vivo* limiting dilution tumor initiation assay. A. Doses of 10, 5 and 2.5 x 10^6 WaGa/tet-CshRNA and WaGa/tet-MUC1shRNA cells were implanted into the left and right flanks, respectively, of NSG mice. **B.** Mice were fed DOX and monitored for tumor formation as shown in the images. **C.** Summary of tet-CshRNA and tet-MUC1shRNA tumors at the indicated doses of injected cells demonstrating that dilution of tet-CshRNA and tet-MUC1shRNA cell number decreases tumor formation in a MUC1-dependent manner.

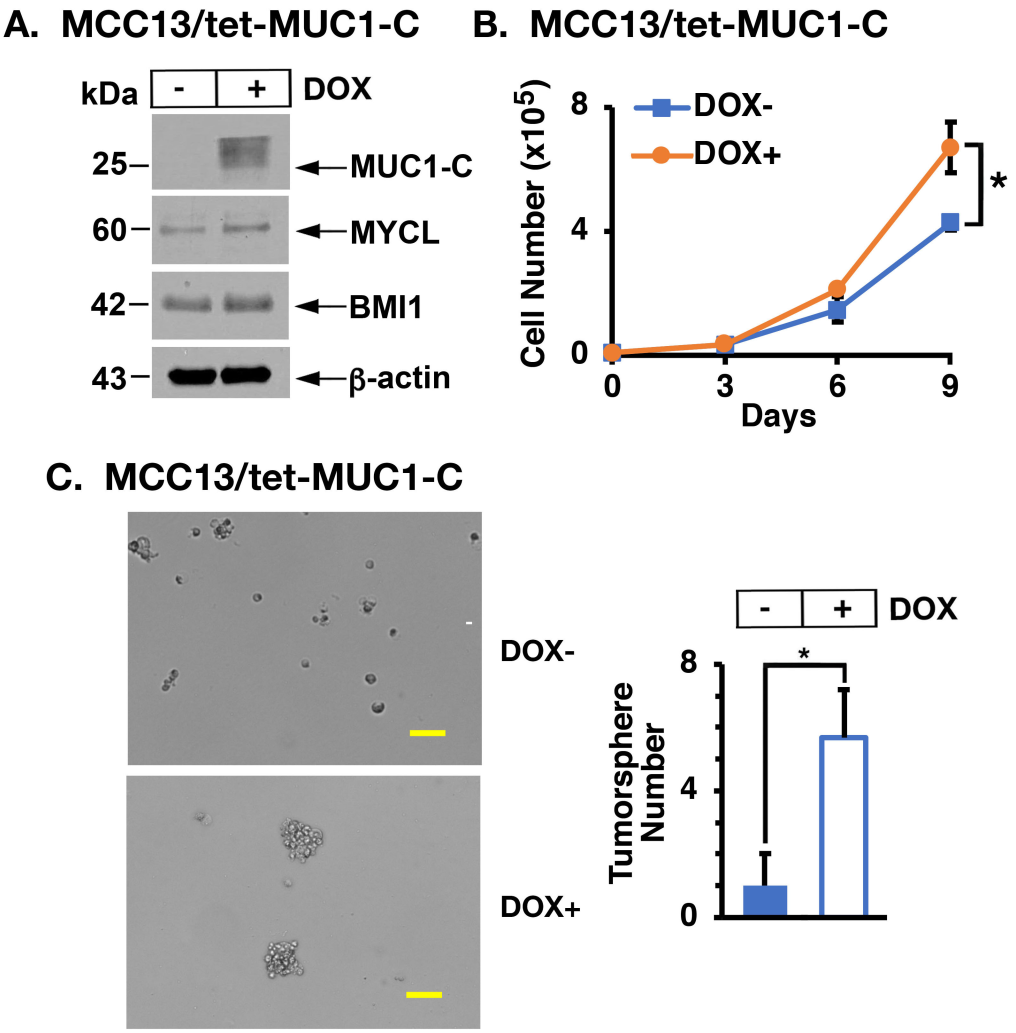

Supplemental Figure S10. Gain-of-function of MUC1-C in MCC13 cells. A-C. MCC13 cells expressing tet-MUC1-C were treated with vehicle or DOX for 12 days. **A.** Lysates were immunoblotted with antibodies against the indicated proteins. **B.** Cells were analyzed for proliferation by trypan blue staining. The results are expressed as the mean±SD of three separate determinations. **C.** Representative images of tumorspheres (left). Bar represents 50 microns. The number of tumorspheres is expressed as the mean±SD of three determinations (right).

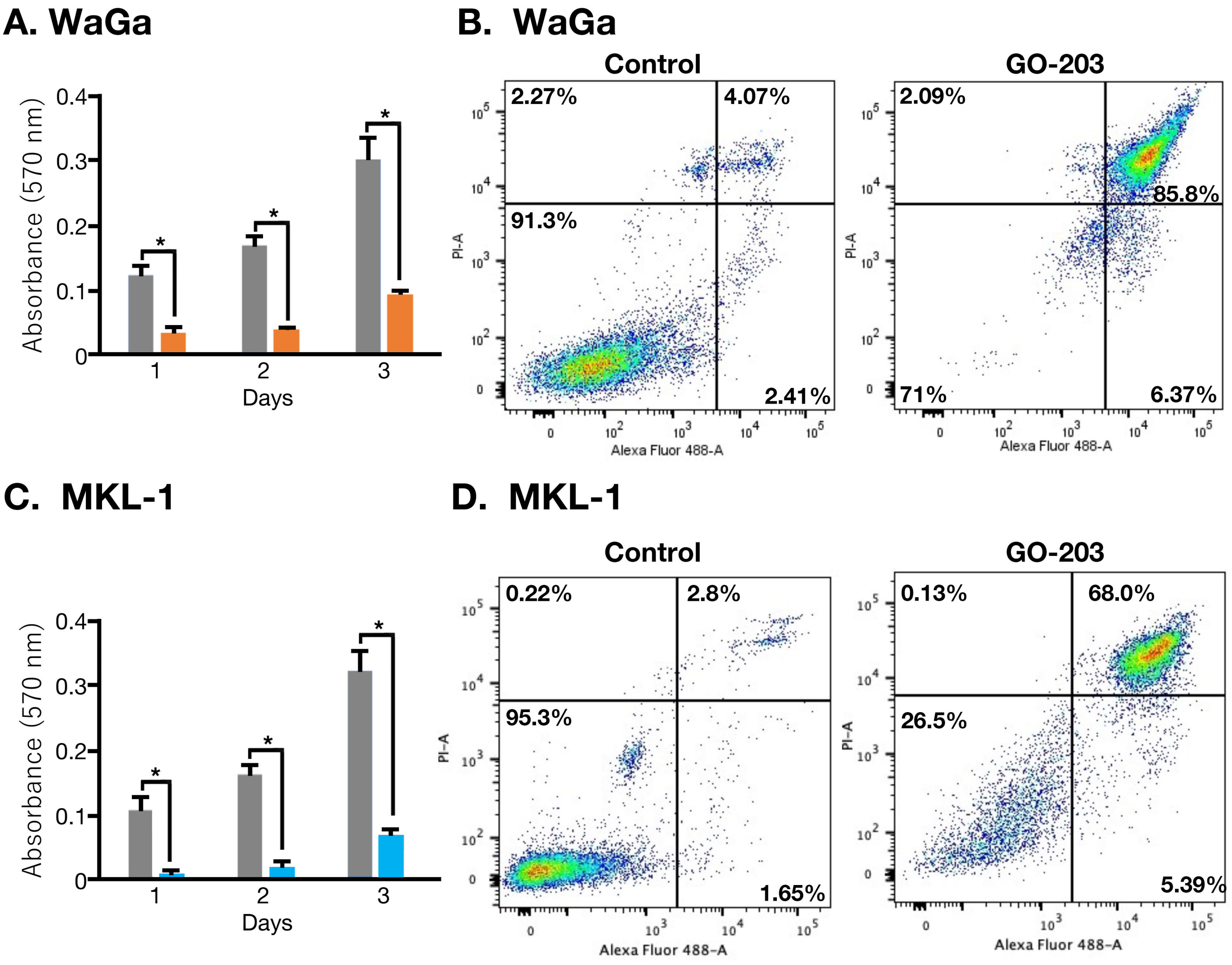

Supplemental Figure S11. Targeting MUC1-C in MCC cells with GO-203 induces apoptosis. A-D. WaGa and MKL-1 cells left untreated (blue bars) and treated with 5 μM GO-203 (orange bars) for the indicated days were analyzed for growth as determined by alamar blue staining (**A,C**). The results are expressed as the mean±SD of three separate determinations. Cells were analyzed for PI and annexin V staining by flow cytometry (**B,D**). The results are representative of 3 separate experiments.

Supplemental Table S1. Primers used for qRT-PCR analysis.

Primer	FWD	REV
**MUC1-C**	AGACGTCAGCGTGAGTGATG	GCCAAGGCAATGAGATAGAC
**SRSF1**	CCTGTTCATCAGGAACGTCG	CGGCCACATACCCACTTTCTA
**SRSF3**	TGGCAACAAGACGGAATTGGA	CAAAGCCGGGTGGGTTTCTA
**SRSF4**	TTAAGGGCTACGGGAAGATCC	ATGCTCAACAATTACTCGCTCA
**BRG1**	CCAAGACCCTGATGAACACC	GGCAGAACAGCAGCACTTT
**BAF60a**	GTATGGGCCAGACAACCATG	ACGAGTCTGGGTATGGATGC
**PBRM1**	AAGAAGAAAGAGCTTGCCAG	TCTCGAGCTTCAAGAACAAC
**β-actin**	GATGAGATTGGCATGGCTTT	CACCTTCACCGTTCCAGTTT
